# The Utility of SOX2 and AGR2 Biomarkers as Early Predictors of Tamoxifen Resistance in ER-Positive Breast Cancer Patients

**DOI:** 10.1155/2021/9947540

**Published:** 2021-09-15

**Authors:** Yomna Zamzam, Yosra Abdelmonem Zamzam, Marwa Aboalsoud, Heba Harras

**Affiliations:** ^1^Department of Pathology, Faculty of Medicine, Tanta University, Tanta, Egypt; ^2^Department of Clinical Pathology, Faculty of Medicine, Tanta University, Tanta, Egypt; ^3^Department of Clinical Oncology and Nuclear Medicine, Faculty of Medicine, Tanta University, Tanta, Egypt

## Abstract

**Background:**

Despite the undeniable benefit of tamoxifen therapy for ER-positive breast cancer patients, approximately one-third of those patients either do not respond to tamoxifen or develop resistance. Thus, it is a crucial step to identify novel, reliable, and easily detectable biomarkers indicating resistance to this drug.

**Objective:**

The aim of this work is to explore SOX2 and AGR2 biomarker expression in the tumor tissue of ER-positive breast cancer patients in combination with the evaluation of serum AGR2 level of these patients in order to validate these biomarkers as early predictors of tamoxifen resistance.

**Methods:**

This study was conducted on 224 ER-positive breast cancer patients. All patients were primarily subjected to serum AGR2 levelling by ELISA and their breast cancer tissue immunostained for SOX2 and AGR2. After 5 years of follow-up, the patients were divided into 3 groups: group 1 was tamoxifen sensitive and groups 2 and 3 were tamoxifen resistant. Time to failure of tamoxifen treatment was considered the time from the beginning of tamoxifen therapy to the time of discovery of breast cancer recurrence or metastases (in months).

**Results:**

SOX2 and AGR2 biomarkers expression and serum AGR2 level were significantly higher in groups 2 and 3 in comparison to group 1, while the relationship between Her2 neu expression and Ki67 index in the 3 different groups was statistically nonsignificant. Lower SOX2 and AGR2 expression and low AGR2 serum levels in the studied patients of groups 2 and 3 were significantly associated with longer time-to-failure of tamoxifen treatment. According to the ROC curve, the combined use of studied markers validity was with a sensitivity of 100%, specificity of 96%, PPV 96%, and NPV 100% (*p* < 0.001; AUC: 0.984).

**Conclusions:**

Integrated use of SOX2 and AGR2 biomarkers with serum AGR2 assay holds a promising hope for their future use as predictive markers for early detection of tamoxifen resistance in ER-positive breast cancer patients.

## 1. Introduction

Estrogen receptor- (ER-) positive breast cancer subtype accounts for 70% of all breast cancers and managed with tamoxifen. It is a drug that competitively inhibits the bonding of estrogen to its receptor [1, 2].

However, more than 50% of advanced estrogen-positive breast cancers are intrinsically resistant to tamoxifen. Besides, almost 40% acquire the resistance during the treatment or may develop recurrence in fifteen years after the five-year tamoxifen therapy [3]. Therefore, identification of early tamoxifen resistance determinants is beneficial to improve efficacy of prognosis and treatment of ER-positive breast cancer patients [4].

Sry-related high-mobility box 2 (SOX2) is a critical transcription factor that has a precious role in different stages of development during the embryonic life and preservation of undifferentiated embryonic stem cells (ESCs) [5]. The relation between Sox2 expression and the clinical aggression of different tumor types, including breast, lung, and prostatic cancers, was observed by several studies [6, 7].

SOX2 is associated with cancer cells resistance development to chemotherapy, radiotherapy, and targeted therapy in different types of human cancers [8]. This could be attributed to its ability to maintain the proliferation of cancer stem cells (CSCs), which are defined as a subpopulation within tumor cells that have stem cell-like properties that endures the treatment and initiates tumor progression [9, 10].

The human anterior gradient-2 (AGR2), an endoplasmic reticulum resident protein, is a member of the protein disulfide isomerase (PDI) gene family [11]. High AGR2 protein levels are detected in serum and/or plasma samples of lung [12], prostate [13], and ovarian [14] cancer patients in comparison to healthy controls suggesting AGR2 as a promising cancer serum biomarker.

Several studies have proved the important function of AGR2 in many cellular activities, such as cellular differentiation, proliferation, transformation, metastasis, and chemotherapy resistance [15, 16]. AGR2 expression was recognized to correlate with dissemination and bad prognosis in breast cancer and reported as a biomarker in prostatic cancer [17, 18].

Despite the mode of action of AGR2 after tamoxifen treatment needs more clarification, Hrstka et al. [11] suggested that AGR2 may significantly affect the development and progression of estrogen-positive breast cancer as well as the response to antihormonal treatment.

The relation between Sox2 and AGR2 expression and breast cancer has been broadly studied. However, the reported results and the prognostic significance of these markers in tamoxifen-treated breast cancer are still conflicting [19, 20].

The aim of this study is to explore SOX2 and AGR2 biomarkers expression in tumor tissue of ER-positive breast cancer patients in combination with evaluation of serum AGR2 level of these patients in order to validate these biomarkers as early predictors of tamoxifen resistance.

## 2. Patients and Methods

### 2.1. Patient Selection

This is a prospective study conducted from January 2011 to January 2015. Selected 260 women were enrolled in this study, 26 patients were deceased, and 10 patients were lost to follow-up. Ultimately, 224 patients were included with 5 years of follow-up. They were admitted to the oncology department of Tanta University Hospitals for breast cancer management. After histopathological confirmation for breast cancer diagnosis, further immunostaining for ER, PR, Her2 neu, and ki67 was done. All consecutive patients with ER-positive immunostaining were selected for this study and all received adjuvant tamoxifen therapy.

Blood samples for serum AGR2 ELISA tests were obtained from the selected ER-positive patients. Moreover, the diagnosed breast cancer paraffin blocks of the same selected patients were subjected to SOX2 and AGR2 immunostaining. The selected patients were followed up for 5 years.

On follow-up, those who developed recurrence or metastasis while on adjuvant tamoxifen were identified. Time-to-failure of tamoxifen treatment was considered the time from beginning of tamoxifen therapy to the time of discovery of breast cancer recurrence or metastases (in months). The patients who are deceased or lost for follow-up were discarded from this study.

This study was approved by the ethics committee of Faculty of Medicine, Tanta University. All included patients provided a written informed consent for the use of their biological specimens for research purposes.

On follow-up, the patients were divided in to three groups, one tamoxifen-sensitive group (group 1) and 2 tamoxifen-resistant groups (groups 2 and 3).

#### 2.1.1. Group 1

This group consisted of 124 ER-positive breast cancer patients who underwent mastectomy or conservative breast surgery promptly after diagnosis of breast cancer. Those selected patients were administered appropriate adjuvant treatment (chemotherapy and/or breast radiotherapy). All started adjuvant tamoxifen therapy, 20 mg per day soon after surgery. These patients did not develop any breast cancer recurrence or metastasis during the 5 years of follow-up. According to this clinical pattern, these breast cancer cases were considered as tamoxifen-sensitive cases.

#### 2.1.2. Group 2

This group consisted of 48 ER-positive patients who failed to respond to primary tamoxifen treatment. These selected patients were diagnosed as ER-positive breast cancer on core biopsy histologically and treated initially with primary tamoxifen treatment and did not have initial surgery because the tumor was locally advanced, and some had advanced age or significant medical comorbidities (as chronic cardiac or lung diseases). From the patients follow-up, some of these tumors continued to progress even on tamoxifen therapy (increase in tumor size clinically and radiologically). They were considered de novo-resistant tumors. If the tumor responded to tamoxifen treatment from the beginning for at least one year (decrease or no change in tumor size clinically and radiologically) then increase in size again after that, these were considered acquired-resistant tumors. Both types of cancer were considered as definite tamoxifen-resistant breast cancer.

#### 2.1.3. Group 3

This group consisted of 52 ER-positive patients who were treated by mastectomy or breast conservative surgery soon after being diagnosed with breast cancer and followed by adjuvant chemotherapy and/or radiotherapy to the breast and adjuvant tamoxifen of 20 mg per day. On follow-up, this group of patients developed breast cancer recurrence or metastasis within 5 years of the primary treatment. These initial tumors could have been either tamoxifen resistant before surgery (de novo resistance) or were initially tamoxifen sensitive, but residual or disseminated breast cancer cells later developed into a tamoxifen-resistant phenotype during tamoxifen treatment (acquired resistance).

### 2.2. Immunohistochemical Staining

Paraffin blocks for selected patients (mastectomy/wide local excision biopsy paraffin blocks for groups 1 and 3 and core biopsy paraffin blocks for group 2) were subjected for SOX2 and AGR2 immunohistochemical staining in Tanta Pathology department. It was performed on 4 *μ*m thick freshly cut tissue sections. Sections were deparaffinized in xylene and rehydrated into PBS through a graded ethanol series. Endogenous peroxidase activity was quenched in 3% hydrogen peroxide in PBS for 15 minutes. Antigen retrieval was performed in citrate buffer pH 6 in 94°C for 20 minutes. The sections were incubated with SOX2-specific rabbit monoclonal antibody (1 : 50, SOX2 clone D6D9, Cell Signaling Technology, Danvers, MA) for 60 min at room temperature and AGR2 rabbit polyclonal antibody (1 : 250 HPA007912, Sigma-Aldrich, St. Louis, MO, USA) overnight at 4°C. A streptavidin-biotin peroxidase detection system was used according to the manufacturer's instructions (Vectastain Ellite ABC Kit, Vector Laboratories, Burlingame, CA, USA). Tissue sections of stomach and small intestine served as external positive controls for SOX2 and AGR2, respectively.

### 2.3. Interpretation and Scoring of Staining

Evaluation of SOX2 and AGR2 immunostaining was done by two pathologists separately. Nuclear SOX2 expression and cytoplasmic AGR2 expression were semi-quantified using a quick score [21], consisting of a four-tiered scoring system for the % of epithelial cells stained positive for SOX2 and AGR2 proteins (1%–10% = 1, 11%–30% = 2, 31%–50% = 3, and >50% = 4) combined with a score for the intensity of staining (0 = no staining, 1 = weak staining, 2 = moderate staining, and 3 = strong staining). The total score was produced by multiplying the score for the proportion of positive cells by the staining intensity category to achieve a final maximum total score of 12 per sample. Scores <5 represented low (1) and scores ≥5 represented high (2) staining for both SOX2 and AGR2 expression.

### 2.4. Measurement of Serum AGR2 by Enzyme-Linked Immunosorbent Assay (ELISA)

It seems that detection of circulating blood biomarkers is a simple rapid method, less invasive technique, and convenient in practice for early screening for tamoxifen resistance in breast cancer patients [16]. Moreover, according to Wayner et al. [22], the combined use of AGR2 ELISA levelling and immunohistochemistry increase the validity of the use of this marker.

Serum samples were prepared by collecting the blood in empty sterile tubes which were left at room temperature for 30 minutes to clot until centrifugation. Samples were centrifuged for 10 min at 4,000 rpm, then the supernatant was transferred to new tubes and analyzed immediately or stored at −20°C until ELISA assay.

Serum levels of AGR2 were measured by means of Sandwich enzyme-linked immunosorbent assay (ELISA). It was carried out as per manufacturer's instructions of the ELISA kit (R&D Systems, Minneapolis, MN, USA). A monoclonal antibody specific for AGR2 had been precoated onto a microplate and incubated with serum samples. After the first washing, an enzyme-linked polyclonal antibody specific for AGR2 was added to the wells to “Sandwich” the AGR2 immobilized on the plate. Following the second wash, a substrate solution was added. The enzyme-substrate reaction was terminated by addition of a sulphuric acid solution. The intensity of color was directly proportional to the concentration of AGR2 in the serum sample. The color reaction was measured spectrophotometrically at a wavelength of 450 nm.

### 2.5. Statistical Analysis

Data were analyzed using IBM SPSS software package version 20.0 (Armonk, NY: IBM Corp). The Kolmogorov-Smirnov test was used to verify the normality of distribution of variables. Comparisons between groups for categorical variables were assessed using Chi-square test (Fisher or Monte Carlo). Mann–Whitney test was used to compare between two groups for not normally distributed quantitative variables, while ANOVA was used for comparing between more than two groups and followed by post hoc test (Tukey) for pairwise comparison. Kruskal–Wallis test was used to compare different groups for not normally distributed quantitative variables. The Kaplan–Meier Survival curve was used. Sensitivity, specificity, area under the receiver operating characteristic (ROC) curve, positive predictive value (PPV), and negative predictive value (NPV) were analyzed. Cut off was choose according to Youden index. Signiﬁcant differences were considered at *p* < 0.05.

## 3. Results

The clinico-pathological characteristics of the three studied groups are illustrated in Table 1. The median age of studied groups was 48, 63.5, and 64 years for group 1, 2, and 3, respectively. The most histopathological type in all studied groups was infiltrating duct carcinoma. Regarding tumor size (T stage), T1 was the most common for groups 1 and 3 (62.9% and 61.5%, respectively); however; T4 was the most common for group 2 (60.4%). For nodal stage, N0 was the most common for groups 1 and 3 (75% and 67.3%, respectively); however, N1 was the most common for group 2 (56.3%). For studied cases, the most common tumor grade was grade 2 in all studied groups. Lympho-vascular invasion was not detected in most studied tumors of group 1 (60.5%) while it was seen mostly in group 2 and 3 tumors (79.2% and 51.9%, respectively). Most patients of the three studied groups were postmenopausal. The median time for tamoxifen therapy failure was 16 and 23 months for groups 2 and 3, respectively. For group 2, de novo resistance to tamoxifen therapy was detected in 31.3% of included patients; meanwhile; acquired resistance was noticed in 68.8% of included patients. All patients in group 3 showed acquired tamoxifen therapy resistance (100%), and the presentation of this therapy failure was in the form of distant metastases (21.2%) or tumor recurrence (78.8%).

All these findings were statistically significant between the three studied groups (*p* value <0.05) except for histopathological type (*p* value: 0.243).

**Figure 1:**
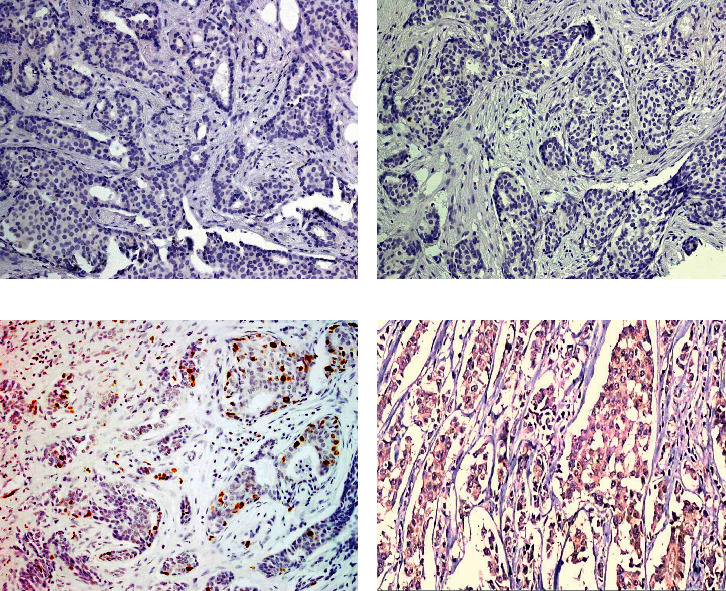
A case of breast carcinoma in group 1 (tamoxifen sensitive) with serum AGR2 level 3 ng/ml showed negative SOX2 expression (a) and negative AGR2 expression (b). Another case of group 1 with serum AGR2 level 7 ng/ml showed low SOX2 nuclear positivity score 3 (c) and low AGR2 cytoplasmic positivity score 2 (d) in malignant cells (X100).

**Figure 2:**
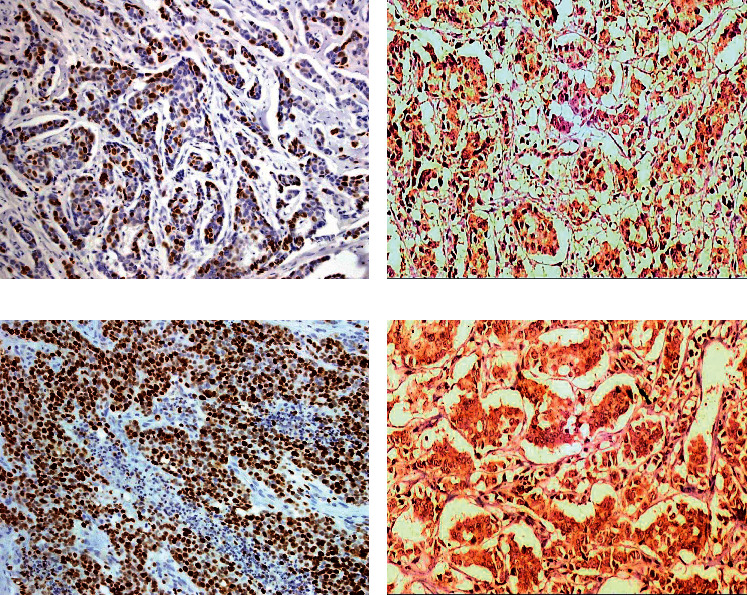
A case of breast carcinoma in group 2 (tamoxifen resistant) with serum AGR2 level 116 ng/ml showed high SOX2 nuclear expression score 9 (a) and high AGR2 cytoplasmic positivity score 8 (b). A case of breast carcinoma in group 3 (tamoxifen resistant) with serum AGR2 level 224 ng/ml showed high SOX2 nuclear positivity score 12 (c) and high AGR2 cytoplasmic positivity score 12 (d) in malignant cells (X100).

SOX2 expression was positive mainly with low positivity (66.1%) in group 1 and mainly with high positivity in groups 2 and 3 (89.6% and 92.3%, respectively). Moreover, AGR2 expression was positive mainly with low positivity (68.5%) in group 1 and mainly with high positivity in groups 2 and 3 (89.6% and 88.5%, respectively). The serum levels of AGR2 were significantly higher in groups 2 and 3 (mean ± SD 141 ± 55.2 and 156.8 ± 44.7, respectively) than group 1 (mean ± SD 12 ± 9.1) (Figures 1 and 2). Her2 neu expression showed only positivity in 29.8%, 36.4%, and 40.4% of groups 1, 2, and 3, respectively. On the other hand, the median Ki67 proliferating index was 15% in group 1, 13% in group 2, and 16.5% in group 3. The relationship between studied markers SOX2, AGR2, and serum AGR2 level was statistically significant in the 3 different groups, while the relationship between Her2 neu expression and Ki67 index in the 3 different groups was statistically nonsignificant as illustrated in Table 2.

**Figure 3:**
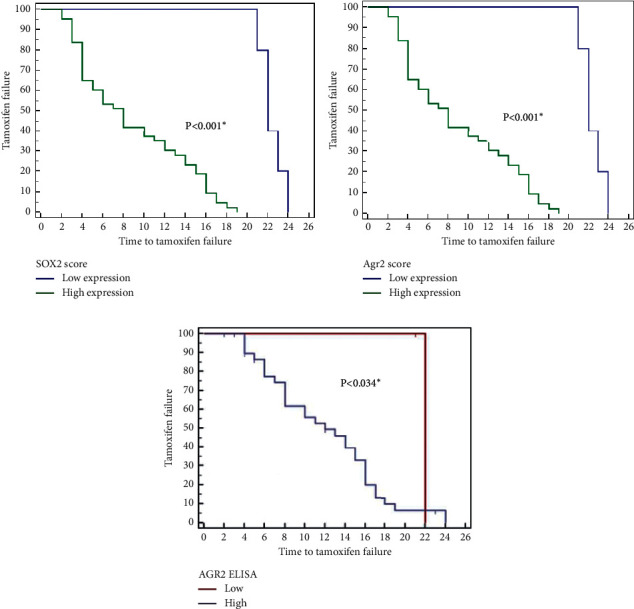
Relationship between SOX2 expression (a), AGR2 expression (b), serum AGR2 level (c), and time-to-failure of tamoxifen treatment (in months) in group 2 (Kaplan-Meier survival curves).

**Figure 4:**
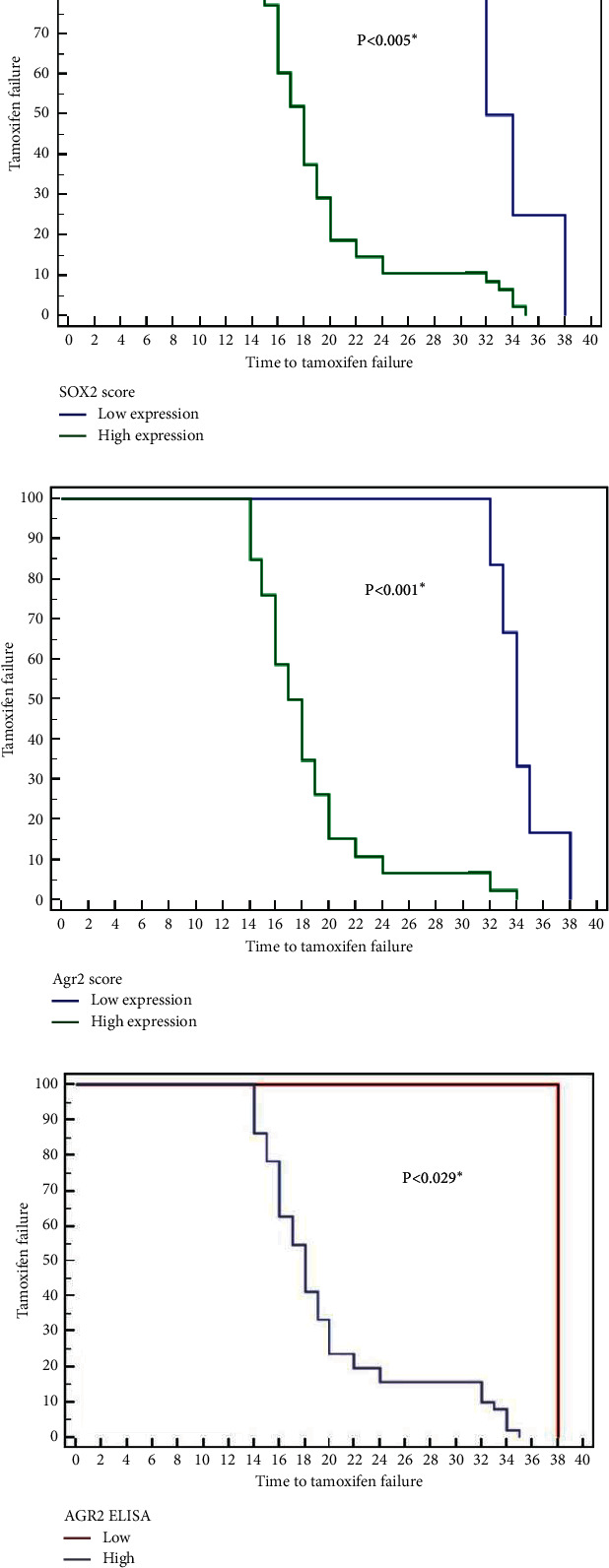
Relationship between SOX2 expression (a), AGR2 expression (b), serum AGR2 level (c), and time-to-failure of tamoxifen treatment (in months) in group 3 (Kaplan-Meier survival curves).

Lower SOX2 and AGR2 expression and low AGR2 serum levels in the studied patients of groups 2 and 3 were significantly associated with longer time-to-failure of tamoxifen treatment. This relation was statistically significant (Figures 3 and 4).

**Figure 5:**
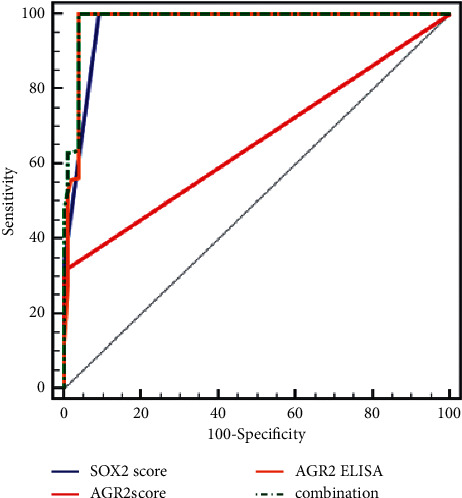
ROC curve for different studied markers to discriminate estrogen-sensitive (group 1) and estrogen-resistant (groups 2 and 3).

The validity of studied markers in discrimination between estrogen-sensitive breast cancer patients and estrogen resistant is illustrated in Table 3 and Figure 5. According to the ROC curve, the validity of SOX2 expression was with sensitivity of 100%, specificity of 91%, PPV 93.2%, and NPV 100% (*p* < 0.001; AUC: 0.970). For AGR2 expression validity was with a sensitivity of 31.45%, specificity of 100%, PPV 100%, and NPV 54.1% (*p* < 0.001; AUC: 0.657). For AGR2 ELISA serum level validity was with a sensitivity of 56.45%, specificity of 96%, PPV 94%, and NPV 64% (*p* < 0.001; AUC: 0.979). The combined use of studied markers validity was with a sensitivity of 100%, specificity of 96%, PPV 96%, and NPV 100% (*p* < 0.001; AUC: 0.984).

## 4. Discussion

Despite the undeniable benefit of tamoxifen therapy for ER-positive breast cancer patients, approximately one-third of those patients either do not respond to tamoxifen or develop resistance, which constitutes a serious clinical problem. Thus, it is a crucial step to identify novel, reliable, and easily detectable biomarkers indicating resistance to this drug [1, 3].

The aim of this study to explore SOX2 and AGR2 biomarkers expression in tumor tissue of ER-positive breast cancer patients in combination with estimation of serum AGR2 level of these patients in order to validate these biomarkers in the early prediction of tamoxifen resistance.

The current study showed significant relationship between age, tumor size (T), nodal status (N), tumor grade, presence of lymphovascular invasion, and time for tamoxifen therapy failure in the three studied groups. Most patients of the three studied groups were postmenopausal. The median time for tamoxifen therapy failure was 16 and 23 months for groups 2 and 3, respectively. Meanwhile, the histopathological type was statistically nonsignificant.

It is important to take into account that our cohort of group 2 was limited to core biopsy only as they did not have initial surgery because the tumor was locally advanced, some had advanced age or significant medical comorbidities (as chronic cardiac or lung diseases). However, the markers study results of this group were close to the results of group 3 (tamoxifen-resistant groups).

Regarding SOX2 expression, it was highly positive in groups 2 and 3 (89.6% and 92.3%, respectively) more than group 1 (66.1%). This finding is in agreement with Piva et al. [9] who stated that tamoxifen-resistant cells were enriched with cancer stem cells and expressed high levels of Sox2 (stem cell marker) compared to breast cancer cells of responders. Besides Sox2 is not just implicated in tumourigenesis but is also involved in the development of resistance to therapy. The increase in the frequency of stem cells and capacity for invasion, suggesting a potential mechanism for the development of resistance to endocrine therapy.

Phillips et al. [23] and Woodward et al. [24] reported that the relevance of the increase in the proportion of cancer stem cells upon tamoxifen treatment is intriguing in the context of the development of tamoxifen resistance in breast cancer patients. Furthermore, Simoes et al. [25] found that Sox2 overexpression activates the Wnt signaling pathway followed by increase of the proportion of breast cancer stem/progenitor cells. In other words, high Sox 2 levels allow the cells to resist the growth inhibitory effects of tamoxifen. The previous findings, with the presence of high Sox2 levels in endocrine-resistant breast cancer, suggest that Sox2 could represent a prognostic factor for tamoxifen resistance development. Besides, Wnt signaling could be an attractive therapeutic target in these patients.

Piva et al. [9] reported also that silencing of the SOX2 gene reduced the size of the stem/progenitor cell population and restored sensitivity to tamoxifen.

Regarding AGR2 expression, it was highly positive in groups 2 and 3 (89.6% and 88.5%, respectively) more than group 1 (68.5%). Besides, the serum levels of AGR2 were significantly elevated in groups 2 and 3 (mean ± SD 141 ± 55.2 and 156.8 ± 44.7, respectively) more than group 1 (mean ± SD 12 ± 9.1). This result is in accordance with Innes et al. [26], Hrstka et al. [27], and Hrstka et al. [11] who reported that breast cancer patients with low AGR2 expression more promptly respond to primary tamoxifen therapy in comparison to tumors with high AGR2 expression.

On the other hand, Hrstka et al. [11] cohort of patients did not show statistical association between AGR2 immunohistochemical expression and response to tamoxifen treatment. This could be attributed to semiquantitative character of the immunohistochemistry staining and very similar levels of AGR2 in more than half of all samples within both estrogen-sensitive and estrogen-resistant groups. Besides, their limited study cohort of patients including only postmenopausal.

Although ER expression itself is the main predictor of response to endocrine therapy, crosstalk between ER and other signaling pathways involved in regulation of cellular growth, survival, stress, and cytokine levels has been mechanistically described in resistance to endocrine agents. Moreover, some reports indicate that AGR2 is involved in the crosstalk between ER and EGFR or PI3K/AKT [28, 29] resulting in endocrine resistance.

Her2 neu expression and Ki67 index were statistically nonsignificant in the 3 studied groups. Elzawahry et al. [30] and Joensuu et al. [31] reported contradictory findings that both Her2 neu and Ki67 significantly overexpressed in tamoxifen-resistant breast cancer cases. This contradiction could be explained by their limiting inclusion only to postmenopausal patients and recurrent breast cancer patients only as tamoxifen resistance cases.

Lower SOX2 and AGR2 expression and low serum AGR2 levels in the studied patients of groups 2 and 3 were significantly associated with longer time-to-failure of tamoxifen treatment. This relation was statistically significant. These data are supported by results of Hrstka et al. [11], Piva et al. [9], and Garczyk et al. [16].

According to the ROC curve, SOX2, AGR2 expression, and serum AGR2 level sensitivity were 100%, 31.45%, and 56.45%, respectively, and the specificity was 91%, 100%, and 96%, respectively (all *p* < 0.001). However, the combined integration of the studied markers increased the validity in order to improve the early detection of tamoxifen resistance with a sensitivity of 100% and specificity of 96% (*p* < 0.001).

The identification of novel predictive biomarkers is essential for an optimal algorithm for adjuvant hormonal therapy in ER-positive breast cancer patients. Our data indicate that decreased expression of SOX2 and AGR2 with low serum level of serum AGR2 clearly related to breast cancer patients who respond and had benefit from tamoxifen-based therapy. On the other hand, high SOX2 and AGR2 expression with high serum level of serum AGR2 may predict a subset of breast cancer patients that are less likely to show adequate tumor growth control or recurrence following tamoxifen therapy. Besides, our findings clearly show the potential usability of serum AGR2 as biomarker for noninvasive early detection of tamoxifen resistance using ELISA.

## 5. Conclusions

Integrated use of SOX2, AGR2 biomarkers with serum AGR2 assay holds a promising hope for their future use as predictive markers for early detection of tamoxifen resistance in ER-positive breast cancer patients. However, larger prospective studies are needed to validate the clinical utility of this panel.

## Figures and Tables

**Table 1:** Clinicopathological characteristics of studied patients in relation to the three studied groups.

	Group 1 (*n* = 124)	Group 2 (*n* = 48)	Group 3 (*n* = 52)	Test of sig.	*p*
*Age (years)*					
Mean ± SD	51.4 ± 12.7	59.9^#^ ± 10	59.5^#^ ± 12.3	*F* = 12.899^*∗*^	<0.001^*∗*^
Median (min.–max.)	48 (28–77)	63.5 (39–77)	64 (36–78)		
*Histological type*					
Infiltrating lobular CA	16 (12.9%)	3 (6.3%)	9 (17.3%)	*χ*2 = 2.832	0.243
Infiltrating duct CA	108 (87.1%)	45 (93.8%)	43 (82.7%)		
*Pathological T stage*					
T1	78 (62.9%)	2 (4.2%)	32 (61.5%)	*χ*^2^ = 132.671^*∗*^	<0.001^*∗*^
T2	33 (26.6%)	12 (25.0%)	16 (30.8%)		
T3	13 (10.5%)	5 (10.4%)	4 (7.7%)		
T4	0 (0.0%)	29 (60.4%)	0 (0.0%)		
*Pathological N stage*					
N0	93 (75.0%)	2 (4.2%)	35 (67.3%)	*χ*^2^ = 80.737^*∗*^	<0.001^*∗*^
N1	24 (19.4%)	27 (56.3%)	14 (26.9%)		
N2	7 (5.6%)	19 (39.6%)	3 (5.8%)		
*Grade*					
I	37 (29.8%)	2 (4.2%)	14 (26.9%)	*χ*^2^ = 17.005^*∗*^	0.002^*∗*^
II	64 (51.6%)	40 (83.3%)	28 (53.8%)		
III	23 (18.5%)	6 (12.5%)	10 (19.2%)		
*Lymph vascular invasion*					
No	75 (60.5%)	10 (20.8%)	25 (48.1%)	*χ*^2^ = 21.797^*∗*^	<0.001^*∗*^
Yes	49 (39.5%)	38 (79.2%)	27 (51.9%)		
*Menopausal status*					
Pre	61 (49.2%)	11 (22.9%)	13 (25.0%)	*χ*^2^ = 14.968^*∗*^	0.001^*∗*^
Post	63 (50.8%)	37 (77.1%)	39 (75.0%)		
*Type of resistance*					
De novo	–	15 (31.3%)	0 (0.0%)	*χ*^2^ = 19.118^*∗*^	<0.001^*∗*^
Acquired	–	33 (68.8%)	52 (100.0%)		
*Presentation for failure*					
Distant metastases	–	–	11 (21.2%)	−	–
Recurrence	–	–	41 (78.8%)		
*Time to tamoxifen failure*					
Mean ± SD.	–	13.4 ± 6.8	23.1 ± 6.2	*U* = 373.50^*∗*^	<0.001^*∗*^
Median (min. – max.)	–	16 (2–24)	23 (14–44)		

*χ*^2^: Chi square test. F: F for ANOVA test, Pairwise comparison bet. Each 2 groups was done using post hoc test (Tukey), U: Mann–Whitney test, *p*: *p* value for comparing between the studied groups, ^#^significant with group 1 and ^*∗*^statistically significant at *p* ≤ 0.05.

**Table 2:** Comparison between the two studied markers SOX2 and AGR2 with Her2neu and ki67 expression in the three studied groups.

	Group 1 (*n* = 124)	Group 2 (*n* = 48)	Group 3 (*n* = 52)	Test of sig.	*p*
*SOX2*					
Negative	42 (33.9%)	0 (0.0%)	0 (0.0%)	*X*^2^ = 41.687^*∗*^	<0.001^*∗*^
Positive	82 (66.1%)	48 (100.0%)	52 (100.0%)		
*SOX2 score*					
Negative	42 (33.9%)	0 (0.0%)	0 (0.0%)	*X*^2^ = 191.275^*∗*^	<0.001^*∗*^
Low	82 (66.1%)	5 (10.4%)	4 (7.7%)		
High	0 (0.0%)	43 (89.6%)	48 (92.3%)		
*AGR2*					
Negative	39 (31.5%)	0 (0.0%)	0 (0.0%)	*X*^2^ = 38.082^*∗*^	<0.001^*∗*^
Positive	85 (68.5%)	48 (100.0%)	52 (100.0%)		
*AGR2score*					
Negative	39 (31.5%)	0 (0.0%)	0 (0.0%)	*X*^2^ = 225.905^*∗*^	<0.001^*∗*^
Low	85 (68.5%)	5 (10.4%)	6 (11.5%)		
High	0 (0.0%)	43 (89.6%)	46 (88.5%)		
*Serum AGR2 level*					
Mean ± SD.	12 ± 9.1	141 ± 55.2	156.8 ± 44.7	*H* = 152.495^*∗*^	<0.001^*∗*^
Median (min. – Max.)	7 (2–32)	130 (4–228)	159.5(9–236)		
*Her2neu*					
Negative	87 (70.2%)	31 (64.6%)	31 (59.6%)	*X*^2^ = 1.932	0.381
Positive	37 (29.8%)	17 (35.4%)	21 (40.4%)		
*Her2neu score*					
0	43 (34.7%)	15 (31.3%)	14 (26.9%)	*X*^2^ = 2.105	0.910
+1	44 (35.5%)	16 (33.3%)	17 (32.7%)		
+2	20 (16.1%)	9 (18.8%)	11 (21.2%)		
+3	17 (13.7%)	8 (16.7%)	10 (19.2%)		
*Ki67 index*					
Mean ± SD.	16.1 ± 9.7	14.7 ± 9.4	17.8 ± 10	*H* = 2.432	0.296
Median (min. – Max.)	15 (3.8–40)	13 (4–38)	16.5 (3.8–40)		

*χ*^2^: Chi square test, *H*: H for Kruskal–Wallis test. ^*∗*^Statistically significant at *p* ≤ 0.05.

**Table 3:** Validity (AUC, sensitivity, and specificity) for SOX2, AGR2 expression, and serum AGR2 level to discriminate between estrogen-sensitive (group 1) and estrogen-resistant (groups 2 and 3).

	AUC	*p*	95% C.I	Cut off^#^	Sensitivity	Specificity	PPV	NPV
SOX2 score	0.970	<0.001^*∗*^	0.948–0.993	≤1	100.0	91.0	93.2	100.0
AGR2 score	0.657	<0.001^*∗*^	0.587–0.728	≤0	31.45	100.0	100.0	54.1
Serum AGR2 level	0.979	<0.001^*∗*^	0.957–1.0	≤9	56.45	96.0	94.6	64.0
SOX2 score + AGR2score + serum AGR2 level	0.984	<0.001^*∗*^	0.968–1.0		100.0	96.0	96.9	100.0

AUC: area under a curve. *p* value: probability value. CI: confidence interval, NPV: negative predictive value, and PPV: positive predictive value. ^*∗*^Statistically significant at *p* ≤ 0.05, ^#^cut off was chosen according to Youden index.

## Data Availability

Hyperlinks are provided for publicly archived datasets analyzed or generated during the study. Help and templates are included with the manuscript.
